# Effect of visual orientation on mu suppression in children: a comparative EEG study with adults

**DOI:** 10.1186/s40101-018-0175-9

**Published:** 2018-06-08

**Authors:** Yuki Nishimura, Yuki Ikeda, Airi Suematsu, Shigekazu Higuchi

**Affiliations:** 1 Graduate School of Integrated Frontier Sciences, Kyushu University, 4-9-1 Shiobaru, Minami-ku, Fukuoka City, Fukuoka Japan; 2 Research Fellow of Japan Society for the Promotion of Science, 4-9-1 Shiobaru, Minami-ku, Fukuoka City, Fukuoka Japan; 3 Faculty of Design, Kyushu University, 4-9-1 Shiobaru, Minami-ku, Fukuoka City, Fukuoka Japan

**Keywords:** Mirror neuron system, Mu suppression, Association model, Growth and development

## Abstract

**Background:**

The human mirror neuron system exists in adults, and even in children. However, a significant, unanswered question in the literature concerns age differences in the effect of visual orientation of human body movements. The observation of actions performed by others is known to activate populations of neural cells called mirror neuron system. Moreover, the power of mu rhythms (8–13 Hz) in the EEG is known to decrease while performing and observing human movements. Therefore, the mu rhythm could be related to the activity of the mirror neuron system. This study investigated the effects of the visual perspective on electroencephalography responses to hand actions in two age groups.

**Methods:**

The participants were 28 elementary school students and 26 university students. Videos of the two hands operating switches were used as stimuli. The electroencephalogram mu rhythm (8–13 Hz) was measured during stimuli presentation as an index of mirror neuron system activity.

**Results:**

Adult participants showed significant mirror neuron system activation under both conditions, although no effect of visual perspectives was observed. On the other hand, children only reacted to egocentric stimuli and not to the others.

**Conclusions:**

These findings confirmed the suggested differences in the activity of the mirror neuron system between different age groups. The demonstration that brain activities related to mirroring change during development could help explain previous findings in the literature.

## Background

The mirror neuron system (MNS) is a cluster of neurons activated during both performance and observation of body movements [1]. They were named by Rizzolatti et al., who first discovered these neurons because of their characteristic of mirroring, which they observed during an experiment on motor neurons of the macaque monkey [2–4]. The existence of MNS in the human brain was suggested by the results of studies using neurophysiological techniques for measuring human brain activity including single-cell recording [5–8]. It was suggested that they might be related to action understanding because the MNS is more sensitive to transitive actions than intransitive actions [9]. Moreover, social cognitive functions such as imitation, empathy, and recognition of facial expression, which are known to be essential functions in building a society, are also related to the MNS [10–15]. Functional magnetic resonance imaging (fMRI) and electroencephalography (EEG) have been used for the measurement of MNS activity in humans because invasive cannot be used due to ethical reasons. EEG mu suppression has been used for this purpose because of its relatively fast response time, reduced environmental restrictions, and the ease of applying to infants and children [16–20]. Braadbaart et al. conducted simultaneous measurement of fMRI and EEG to test the reliability of mu wave suppression as a measure of MNS activity. They concluded that mu wave is a possible measure of MNS activity. However, contamination with other brain regions has been indicated [21–24].

The MNS can be found even in children. It is well known that a developing child shows imitating behavior, and this is thought to be the result of the emerging MNS [25]. Many studies have been conducted with autism spectrum disorder (ASD) children to investigate the relationship between MNS dysfunction and ASD [19]. However, less attention has been given to the MNS of typically developing children. Moreover, most participants used to test the existence of the MNS, and its characteristics have ranged from infancy to kindergarten age groups [26], or those past their adolescence.

Past studies have suggested long- and short-term modulation of MNS activity [27–29]. One possible explanation of the plasticity of MNS is association learning, in which associations between somatosensory and visual information are repeatedly established during the development and form mirror-like responses [30]. Therefore, a study comparing typically developing elementary school students and typically developing adults could contribute to understanding the process of acquiring and refining the MNS through adaptation and development.

Many studies have been conducted to determine the distinguishing characteristics of the MNS, and several factors that affect MNS activity have been reported. The spatial-visual orientation of stimuli is one of the basic factors that could change this activity; however, no unified view of the effect has been established to date. There are several studies using different neurophysiological methods that have reported greater mirroring activity resulting from an egocentric perspective compared to an allocentric perspective [31–34]. On the other hand, there are also studies that have reported opposite results indicating a stronger effect of allocentric stimuli on eliciting MNS activity [35, 36]. Moreover, Burgess et al. found no difference between the two perspectives. They concluded that the participants’ MNS were flexible enough to act at the same level even when the perspective changed [37]. However, all these results have been obtained from adult participants. To our knowledge, there is no study that has examined the effect of visual orientation on the MNS of children. Moreover, no study has directly compared the effect of visual orientation between children and adults.

The aim of this study was to determine the effect of the viewer’s perspective on mu desynchronization in children. We designed an experiment to directly compare elementary school children and adults to clarify the effect of visual perspective on children’s mirroring activity. Social brain activity and biological features dramatically change during adolescence [38]. Therefore, it is worthy not to include adolescent participants to highlight the age difference. Children have completed less associative learning than adults. Therefore, we expected a greater mu wave power attenuation in children while observing egocentric stimuli, because associative learning in children would be conducted mainly through self-oriented sensorimotor events. The event-related desynchronization of the mu wave was measured in children and adults because of its convenience and the low degree of invasiveness.

## Methods

### Participants

Advertisements recruited healthy elementary school students (*N* = 28; 14 boys and 14 girls; aged 8 to 12 years, mean age 9.8 ± 1.3 years) and healthy university students (*N* = 26; 13 men and 13 women; aged 19 to 30 years, mean age 22.7 ± 1.9 years) as participants. All the participants or their parents reported that they were right-handed, and had normal or corrected-to-normal vision. They had not diagnosed as having developmental disorder ever since and were naïve to the purpose of the experiment. The participants or their parents were informed that the privacy of the participants would be protected. Furthermore, the participants or their parents gave their written informed consent before participation in the study.

The procedure of the study was approved by the Ethics Committee of Kyushu University. The study was conducted according to the principles of the Declaration of Helsinki.

### Equipment

The study was conducted in an acoustically and electrically sealed room. A 64-channel EEG was recorded using an EEG amplifier (Net Amps 200 64-channel EEG amp., EGI) and a sensor net (Hydrocel Geodesics Sensor Net, EGI) with acquisition software (Net Station 4.3.1, EGI) running on a computer (Power Book G4, Apple Inc.). The amplifier’s hardware low-pass filter was set to 100 Hz, and electrode impedance was maintained to remain under 70 kΩ as suggested by EGI. The data was sampled at 250 Hz. Stimuli were delivered with Presentation Ver. 18.1 (NBS Inc.) and an LCD (E2351VR-BN, LG Electronics) refreshing on 60 Hz using Cz as reference electrode. The display was placed at 0.7 m ahead of the participant seated in the middle of the room.

### Stimuli

**Fig. 1:**
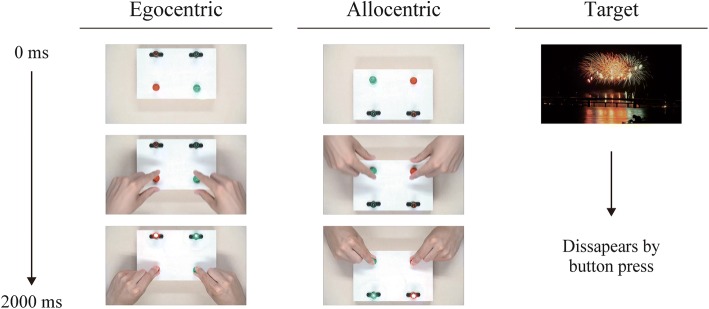
Example of stimuli presented during the EEG recording. Initially, no hands were displayed in the video. After approximately 1 s, the hands appeared holding switches and operating them to turn on the lights. Left: In the egocentric condition, the hands appeared from the bottom of the screen so that the action was observed as the person’s own action. Right: In the allocentric condition, the spatial orientation was rotated by 180°

Video clips that were each 2 s in length (1280 × 720 px, 60 fps, visual angle 16°) were repeatedly presented with a black background in egocentric and allocentric perspectives to elicit brain activity. The egocentric video shows two hands appearing from the bottom of the screen to operate the switch from the neutral position to turn lights on. The video for the allocentric condition was a 180°-rotated version of the egocentric stimulus, ensuring identical physical properties among the two conditions (Fig. 1). The video clips were developed based on previous findings of pronounced effects of hand movement and hand-operated tools on MNS activity [39, 40]. A static picture of fireworks was presented as the target for a button-pressing request that was added to maintain the participants’ attention.

Stimuli presentation was conducted in three blocks of videos consisting of 45 stimuli (135 in total; 60 of each condition, egocentric and allocentric; and 15 target pictures) interrupted by two short breaks. A white fixation cross on a black background was displayed for 1000–2000 ms before the video presentation. Target pictures were set to disappear simultaneously when the button was pressed. After the video playback, a black screen was inserted for an interval of 2 s. The order of presentations was randomized.

### Procedure

After the sensor net was applied, the participants sat comfortably on a chair facing the monitor placed on a desk. EEG mu wave event-related desynchronization (ERD) of the onset of stimuli was measured during the presentation of stimuli. Several previous experiments and meta-analyses including spontaneous recording of fMRI and EEG have indicated that mu wave attenuation is a reliable measure of MNS activity in adults, children, and infants [21, 41–46]. Participants were requested to look at the center of the display during the experiment and refrain from making any eye or body movements as far as possible.

### EEG analysis

First, raw EEG data were imported into the EMSE Suite Ver. 5.5.2 (Source Signal Imaging Inc.) and were re-referenced to the linked left and right mastoids. The data were filtered by an IIR band-pass filter (high end 70 Hz, low end 0.5 Hz, filter order 3). Trials containing potential(s) greater than ± 70 μV that originated from artifacts were excluded by manual inspection (mean number of accepted trials per condition 48.17 ± 7.73). After the selection, six participants (three adults and three children) were omitted from further analysis due to insufficient numbers of acceptable trials (≦ 20 trials per condition).

Next, mu ERD was calculated using R Ver. 3.4.4 (R Core Team) [47] by using the following procedure: (1) According to the study by Oberman et al., [19], the average mu wave power, 8~13 Hz of the baseline period (− 700 to − 500 ms before stimulus onset) was calculated by the Hanning window and fast Fourier transform (FFT). (2) The suppression rate was calculated by ([*A* − *R*]/*R*) × 100 with *A* being the average mu power during 1100 to 2000 ms after stimulus onset (from the onset of movement in the video to the end) and *R* being mu power during the baseline period. (3) These steps were repeated for all stimuli presentation in each condition.

**Fig. 2:**
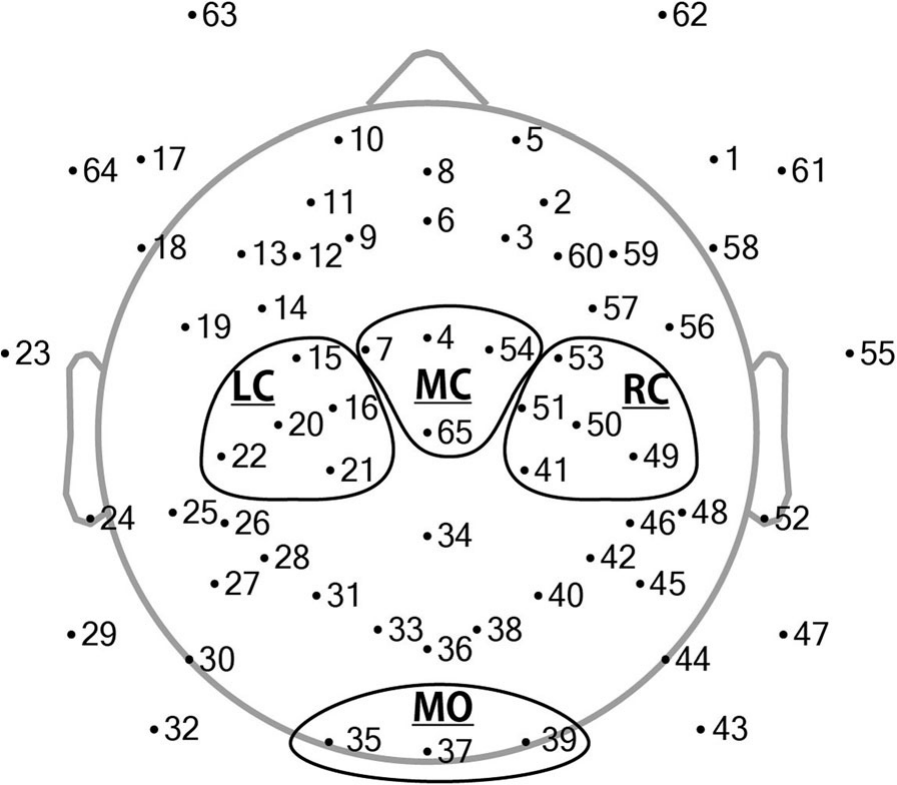
Sensor layout of the EEG cap used in the current study. Each dot represents one of 65 channels. Channels included in the four ROIs are circled

Finally, ERDs of the channels were averaged across the regions of interest (ROI) to improve the reliability of the data (Fig. 2). The left central (LC) region included channels 15, 16, 20, 21, and 22; the mid-central (MC) region included channels 4, 7, 54, and 65 (Cz); and the right central (RC) region included channels 41, 49, 50, 51, and 53. The mid-occipital (MO) regions consisting of channels 35, 37, and 39 were also added to the analysis to compare occipital activity.

R version 3.4.4 (R Core Team) [47] was used for the analysis. Kolmogorov-Smirnov test operated on the ERD confirmed that the data was normally distributed (*D* = 0.051, *p* = 0.28). Firstly, a one-sample *t* test was conducted on ERD data at each ROI to check whether significant mu suppression occurred during stimuli presentation relative to the baseline. Secondly, in order to take within-subject correlations originated from the repeated measurements into account while testing the effect of view point on central and occipital ERD as target variable, we used linear mixed-effect model (LMM: age-group, condition and ROI as fixed effects, and “subject” as random effect) [48, 49]. The values were compared using mixed-design analysis of variance (ANOVA) for the fitted model with Satterthwaite’s method for the estimation of the denominator degree of freedom [48]. We first conducted mixed-design three-way ANOVA with age group (children and adults), anterior-posterior position (central and occipital), and condition (egocentric and allocentric) as fixed effects to test the effect of the viewpoint on central and occipital ERD. Since interactions were observed between all three factors, additional ANOVAs were conducted separately in central and occipital regions. In the central region, mixed-design three-way ANOVA (2 × age group; 3 × ROI; and 2 × condition) was conducted to test the effect of viewpoint on ERD. In the occipital region, a mixed-design two-way ANOVA (2 × age group and 2 × condition) was conducted on alpha wave ERD. The significance level for all the tests was set at 0.05.

## Results

**Fig. 3:**
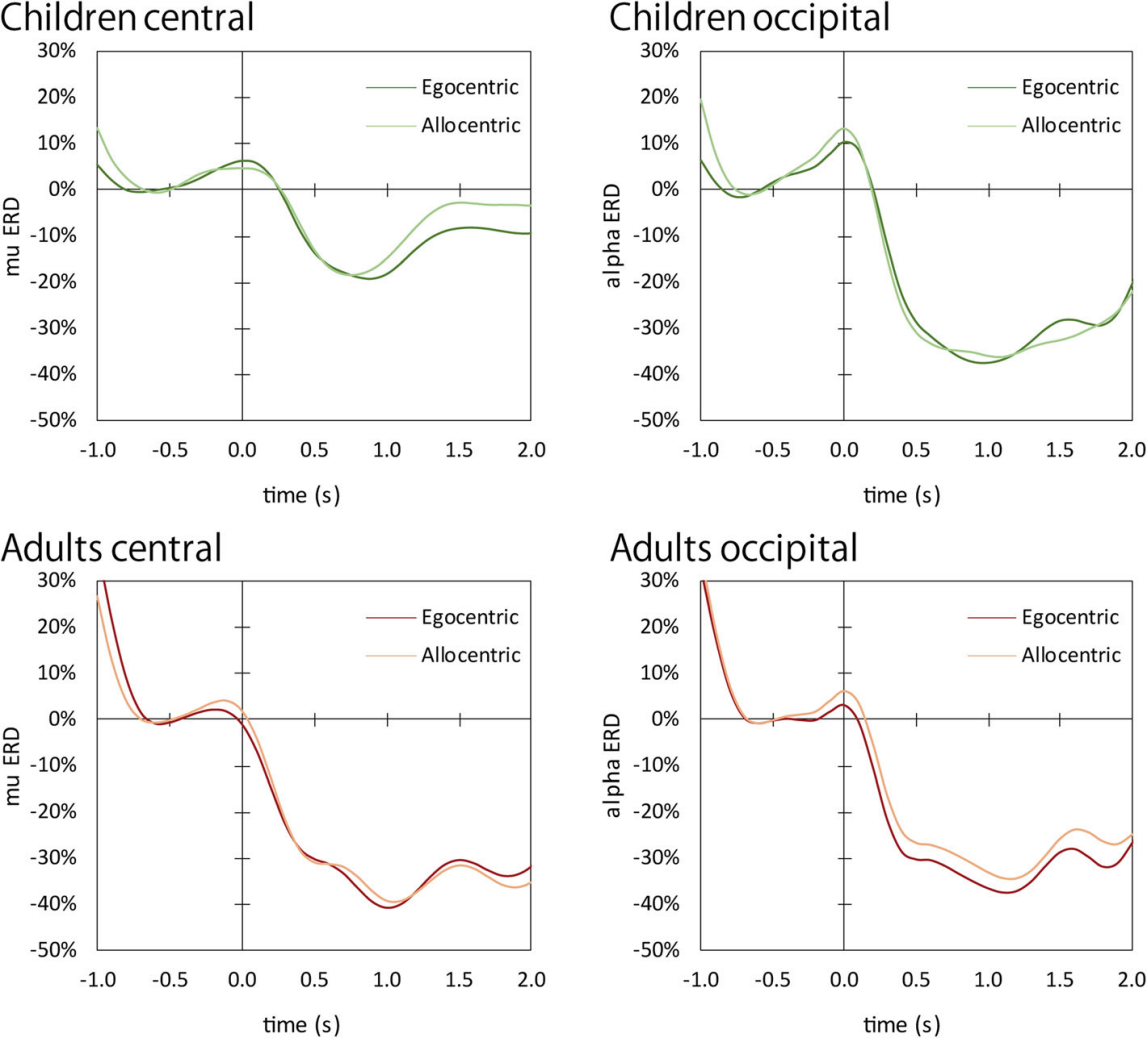
Grand-average mu and alpha event-related desynchronization recorded in children and adults. The darker line represents the ERD waveform for the egocentric condition, and the lighter line represents the waveform for the allocentric condition

**Fig. 4:**
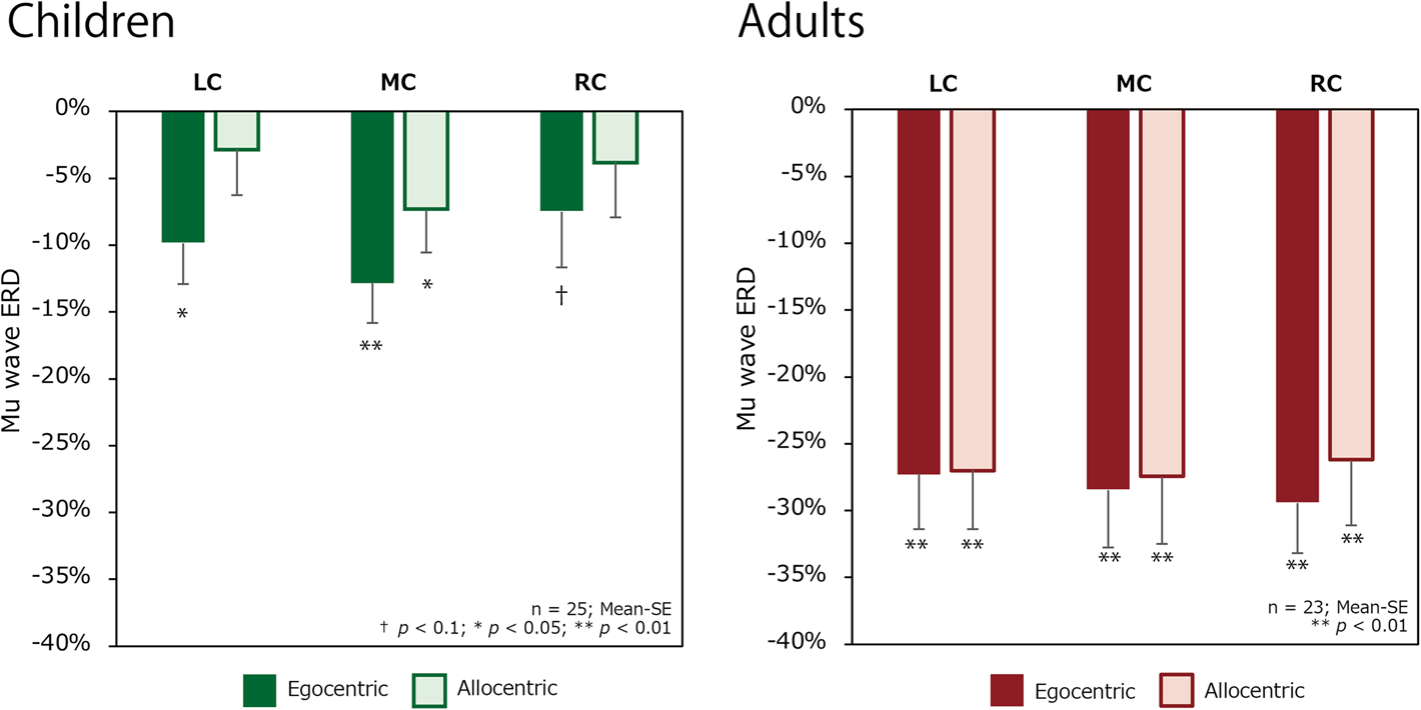
Mean mu wave ERD difference over three ROIs between egocentric and allocentric conditions **a** for children and **b** for adults (LC, MC, and RC). The filled bar represents the egocentric condition, and the outlined bar represents the allocentric condition. Error bars show ± 1 standard errors. Significant differences from zero are indicated. ^†^*p* < 0.1; *p < 0.05; **p < 0.01. It is assumed that brain activation is greater in regions where a stronger ERD is observed

Grand averaged mu and alpha ERD waveforms are shown in Fig. 3. Figure 4 shows mu suppression of the two age groups for each condition. One-sample *t* tests showed mu event-related suppression by egocentric stimuli to be significantly different from zero over all three central ROIs for both children (LC: *t*(24) = − 3.234, *p* = 0.004; MC: *t*(24) = − 4.251, *p* < 0.001; RC: *t*(24) = − 1.764, *p* < 0.090) and adults (LC: *t*(22) = − 6.938, *p* < 0.001; MC: *t*(23) = − 6.678, *p* < 0.001; RC: *t*(23) = − 7.325, *p* < 0.001). This indicated a significant mu power attenuation during stimuli presentation relative to the baseline. The mu ERD of allocentric stimuli was not significantly different from zero for children’s LC (*t*(24) = − 0.823, *p* = 0.419) and RC (*t*(24) = − 0.933, *p* = 0.360), whereas the MC of children (*t*(24) = − 2.309, *p* = 0.030) and adults (LC: *t*(22) = − 7.062, *p* < 0.001; MC: *t*(23) = − 6.762, *p* < 0.001; RC: *t*(23) = − 6.747, *p* < 0.001) indicated significant difference from zero.

**Fig. 5:**
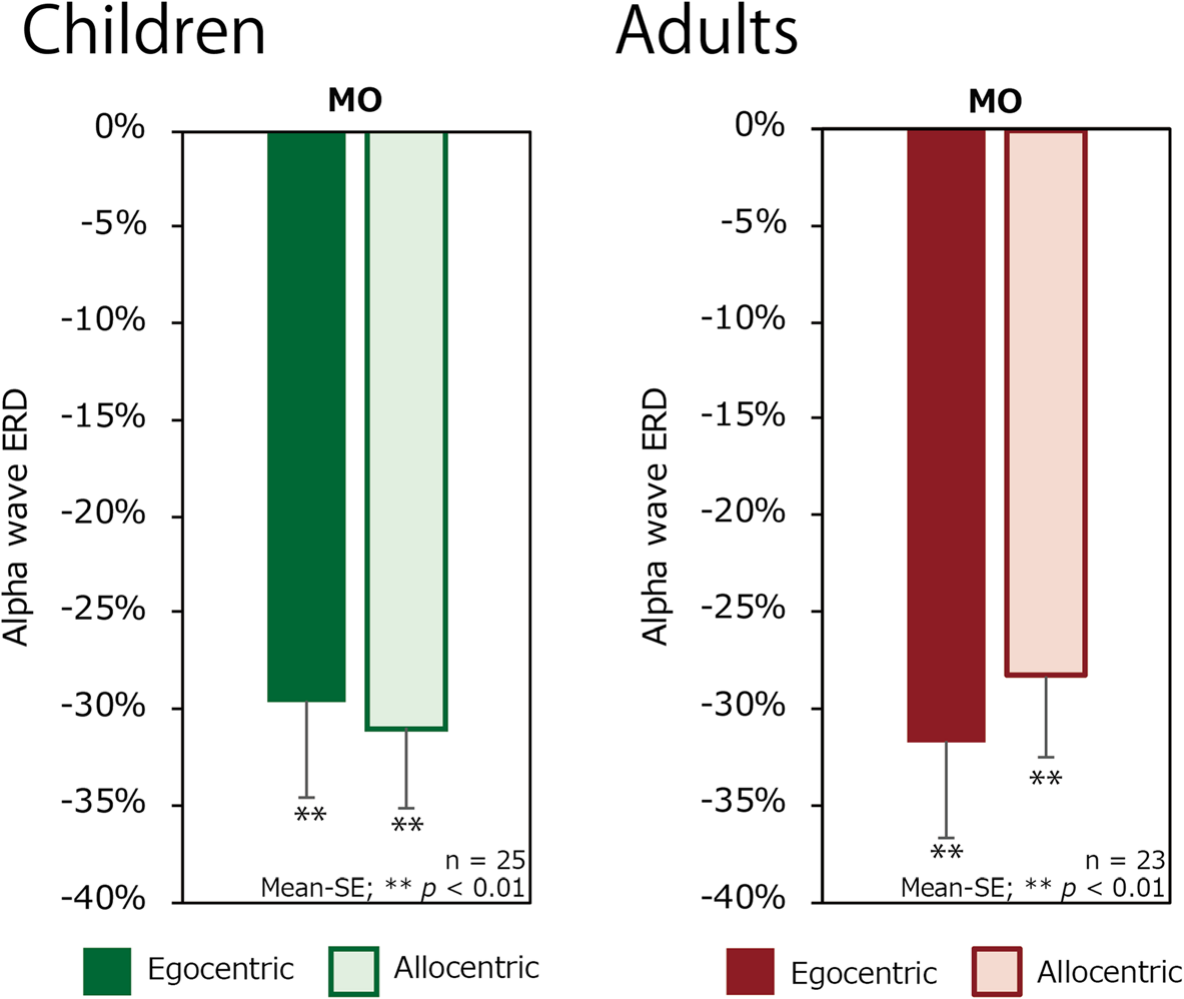
Alpha wave ERD difference between conditions (egocentric and allocentric) **a** for children and **b** for adults over the mid-occipital (MO) region. The filled bar represents the egocentric condition, and the outlined bar represents the allocentric condition. The error bars display standard errors. Significant differences from zero are indicated. **p < 0.01

Figure 5 shows the alpha suppression of both age groups for each condition. One-sample *t* tests indicated significant alpha suppression from zero around the MO region for all stimuli in both children (egocentric: *t*(24) = − 5.906, *p* < 0.001; allocentric: *t*(24) = − 7.4332, *p* < 0.001) and adults (egocentric: *t*(22) = − 4.710, *p* < 0.001; allocentric: *t*(22) = − 3.997, *p* < 0.001).

The conditional and marginal coefficient of determination for the model fitted by LMM was *R*^2^_c_ = 0.883 and *R*^2^_m_ = 0.209 for the first model (group, condition and anterior-posterior position as fixed effects, and “subject” as random effect), *R*^2^_c_ = 0.927 and *R*^2^_m_ = 0.301 for the model for central regions (group, condition and ROI as fixed effects, and “subject” as random effect), and *R*^2^_c_ = 0.827 and *R*^2^_m_ = 0.002 for the model for occipital region (group and condition as fixed effects, and “subject” as random effect). Conditional *R*^2^ is interpreted as variance explained by both fixed and random factors while marginal *R*^2^ is interpreted as variance explained only by the fixed factors [50]. Importantly, mu ERD variance was explained by the fixed factors for about 30% while alpha ERD at occipital region was mainly explained by the random factors and not by the fixed factors at all.

The first ANOVA showed a significant interaction of group, anterior-posterior position, and condition (*F*(1, 234.2) = 6.462, *p* = 0.012). Therefore, we separated the dataset of central and occipital regions for the further analysis. The ANOVA on central ROIs showed a significant main effect of age group (*F*(1, 46) = 25.222, *p* < 0.001) and a trend of the group × condition interaction (*F*(1, 46) = 3.236, *p* = 0.079) on mu ERD. Based on our hypothesis that effect of visual perspective differs between groups, we further investigated this interaction by examining the effect of condition by calculating pairwise least squares means differences [48]. Figure 4 shows condition difference for children (*t*(46.0) = 2.16, *p* = 0.036) suggesting that the mu wave ERD of children was greater in the egocentric condition, whereas there was no significant difference for adults (*t*(46.0) = − 0.421, *p* = 0.676). In contrast, an ANOVA conducted on alpha ERD measured around MO showed neither a significant main effect (*F*(1, 46) = 0.175, *p* = 0.678), group (*F*(1, 46) = 0.001, *p* = 0.973), nor an interaction (*F*(1, 46) = 0.977, *p* = 0.328) (Fig. 5). This suggests that the attentional level which is reflected in the occipital alpha was kept at the same level in both groups at both conditions.

## Discussion

The current study was designed to investigate whether there was an effect of differences in visual orientation on children’s mu wave oscillation compared to adults when observing identical hand movements from two different viewpoints. Results indicated that only the children’s EEG response was affected by the change in visual orientation, whereas there was no significant effect of the perspective on adults’ mu wave ERD. Additionally, alpha wave ERD over the occipital region did not show any significant main effect nor interaction related to the condition, which is indicative of similar occipital cortex activity regardless of the condition or the age group.

### Children

There was no significant mirroring activity in children in response to allocentric stimuli, which could be attributed to the degree of associative learning in children. In addition to the “adaptive model” of MNS, a developmental perspective on the MNS has been suggested by the relatively new “association model” [30]. This model explains the acquisition of MNS and its plasticity through sensory-motor association learning during development. In this model, associative learning, for example, can take place through the visual-motor integration of a person’s own hand movements [51, 52]. Therefore, associative learning could later result from egocentric and allocentric perspectives. A lower level of learning is expected to result in less mirroring activity. There is some evidence that age modulates the effect of motor learning during the elementary school period [53], which is suggestive of an ongoing process of association learning during that period.

Another possible explanation of these results is spatial perspective taking ability. It is known that this psychological ability is acquired during development along with the development of the frontal lobe function [54, 55]. These studies suggested that the egocentric view is more familiar to us whereas the allocentric view requires additional frontal intervention, which is insufficiently developed in children. Also, during spatial perspective taking tasks, somatosensory, premotor, and supplementary motor brain regions overlap with brain regions employed by the MNS [56–58], which is suggestive of a strong relationship between perspective taking and mirroring neural activity. Thus, the results of this study are explained by spatial perspective taking ability, as well as by development.

### Adults

There has been much interest in the effect of the visual perspective on MNS activity, and a large number of studies have been conducted on this topic. Results of different studies conducted on adult participants have suggested the superiority of the egocentric perspective in eliciting human and primate MNS activity [31–34], which are inconsistent with the results of this study. Moreover, our results on adults are inconsistent with studies that have reported the superiority of the allocentric perspective [35, 36]. Therefore, the results of this study must be interpreted with care. There are, however, certain differences between previous and the current study, including the number of hands that were used as stimuli. Observing bimanual movements, for example, might require different patterns of motor coding.

The new model that takes the developmental aspect of MNS into consideration predicts that mirroring response should increase for actions with strongly established associations [30]. As suggested in the literature by Burgess et al., our adult participants might have already completed their association learning. As a result, they might have lost the novelty value of allocentric stimuli, which might have eliminated the differential effect of experimental conditions on adults [37].

### Limitations and future study

The mu wave frequency band was set to 8–13 Hz regardless of the age group, which is the same bandwidth used in previous studies [42, 59–61]. However, the peak alpha wave differs according to age. Mean alpha peak frequency is approximately 9.4 Hz for participants in the current study [62]. Although alpha wave attenuation and mu wave attenuation have different sources as suggested by the present study, reconsideration of the frequency band of children’s mu wave may provide a more robust result. Furthermore, certain studies have suggested that mu suppression could include alpha suppression and its reliability has to be considered carefully [22–24, 63]. Additional study with the same design but using different neurophysiological methods is also required in the future. In addition, collecting individual’s autism-related quotient may aid to assess the putative relation between ASD and MNS activity in the future study.

## Conclusion

In conclusion, this study indicated that children’s MNS activity could be affected by the visual orientation of a stimulus. Along with the results of data on adults, our findings contribute to the understanding of developmental aspects of the MNS. It is suggested that further research using multistep age groups ranging from childhood to adolescence and adulthood is required.

## Abbreviations

ANOVA: Analysis of variance
EEG: Electroencephalogram
LMM: Linear mixed-effect model
MNS: Mirror neuron system

## References

[1] Iacoboni M (2009). Imitation, empathy, and mirror neurons. Annu Rev Psychol.

[2] Di Pellegrino G, Fadiga L, Fogassi L, Gallese V, Rizzolatti G. Understanding motor events: a neurophysiological study. Exp Brain Res. 1992;91:176–80. Available from: http://link.springer.com/10.1007/BF00230027. 10.1007/BF00230027 1301372

[3] Gallese V, Fadiga L, Fogassi L, Rizzolatti G. Action recognition in the premotor cortex. Brain. 1996;119(Pt 2):593–609. Available from: 10.1093/brain/119.2.593. 10.1093/brain/119.2.593 8800951

[4] Rizzolatti G, Fadiga L, Gallese V, Fogassi L. Premotor cortex and the recognition of motor actions. Cogn. Brain Res. 1996;3:131–41. Available from: 10.1016/0926-6410(95)00038-0. 10.1016/0926-6410(95)00038-0 8713554

[5] Fadiga L, Fogassi L, Pavesi G, Rizzolatti G (1995). Motor facilitation during action observation: a magnetic stimulation study. J Neurophysiol.

[6] Mukamel R, Ekstrom AD, Kaplan J, Iacoboni M, Fried I. Single-neuron responses in humans during execution and observation of actions. Curr. Biol. 2010;20:750–6. Available from: 10.1016/j.cub.2010.02.045. 10.1016/j.cub.2010.02.045 PMC2904852 20381353

[7] Strafella a P, Paus T (2000). Modulation of cortical excitability during action observation: a transcranial magnetic stimulation study. Neuroreport.

[8] Babiloni C, Del Percio C, Vecchio F, Sebastiano F, Di Gennaro G, Quarato PP, et al. Alpha, beta and gamma electrocorticographic rhythms in somatosensory, motor, premotor and prefrontal cortical areas differ in movement execution and observation in humans. Clin. Neurophysiol. 2016;127:641–54. Available from: 10.1016/j.clinph.2015.04.068. [cited 2015 Sep 10]. 10.1016/j.clinph.2015.04.068 26038115

[9] Iacoboni M, Molnar-Szakacs I, Gallese V, Buccino G, Mazziotta JC, Rizzolatti G. Grasping the intentions of others with one’s own mirror neuron system. PLoS Biol. 2005;3:e79. Available from: 10.1371/journal.pbio.0030079. [cited 2015 May 27]. 10.1371/journal.pbio.0030079 PMC1044835 15736981

[10] Carr L, Iacoboni M, Dubeau M-C, Mazziotta JC, Lenzi GL. Neural mechanisms of empathy in humans: a relay from neural systems for imitation to limbic areas. Proc Natl Acad Sci USA. 2003;100:5497–502. Available from: 10.1073/pnas.0935845100. 10.1073/pnas.0935845100 PMC154373 12682281

[11] Ferrari PF, Gallese V, Rizzolatti G, Fogassi L. Mirror neurons responding to the observation of ingestive and communicative mouth actions in the monkey ventral premotor cortex. Eur. J Neurosci. 2003;17:1703–14. Available from: 10.1046/j.1460-9568.2003.02601.x. 10.1046/j.1460-9568.2003.02601.x 12752388

[12] Gallese V, Goldman A. Mirror neurons and the simulation theory of mind-reading. Trends Cogn. Sci. 1998;2:493–501. Available from: 10.1016/S1364-6613(98)01262-5. 10.1016/s1364-6613(98)01262-5 21227300

[13] 1. Molenberghs P, Cunnington R, Mattingley JB. Is the mirror neuron system involved in imitation? A short review and meta-analysis. Neurosci Biobehav Rev [Internet]. 2009;33:975–80. Available from: http://linkinghub.elsevier.com/retrieve/pii/S014976340900044X. 10.1016/j.neubiorev.2009.03.010 19580913

[14] Rizzolatti G, Sinigaglia C (2010). The functional role of the parieto-frontal mirror circuit: interpretations and misinterpretations. Nat. Rev. Neurosci.

[15] Woodruff CC, Martin T, Bilyk N (2011). Differences in self- and other-induced Mu suppression are correlated with empathic abilities. Brain Res.

[16] Pineda JA. The functional significance of mu rhythms: Translating “seeing” and “hearing” into “doing.” Brain Res Rev [Internet]. Department of Cognitive Science and Neuroscience, University of California, San Diego, San Diego, CA 92037-0515, United States; 2005;50:57–68. Available from: 10.1016/j.brainresrev.2005.04.005. 10.1016/j.brainresrev.2005.04.005 15925412

[17] Désy MC, Lepage JF (2013). Skin color has no impact on motor resonance: evidence from mu rhythm suppression and imitation. Neurosci Res.

[18] Marshall PJ, Young T, Meltzoff AN (2011). Neural correlates of action observation and execution in 14-month-old infants: an event-related EEG desynchronization study. Dev Sci.

[19] Oberman LM, McCleery JP, Hubbard EM, Bernier R, Wiersema JR, Raymaekers R (2013). Developmental changes in mu suppression to observed and executed actions in autism spectrum disorders. Soc Cogn Affect Neurosci.

[20] Warreyn P, Ruysschaert L, Wiersema JR, Handl A, Pattyn G, Roeyers H (2013). Infants’ mu suppression during the observation of real and mimicked goal-directed actions. Dev Sci.

[21] Braadbaart L, Williams JHG, Waiter GD (2013). Do mirror neuron areas mediate mu rhythm suppression during imitation and action observation?. Int. J Psychophysiol.

[22] Hobson HM, Bishop DVM. Mu suppression – A good measure of the human mirror neuron system? Cortex [Internet]. 2016 [cited 2016 Apr 18];82:290–310. Available from: 10.1016/j.cortex.2016.03.019. 10.1016/j.cortex.2016.03.019 PMC4981432 27180217

[23] Fox NA, Bakermans-Kranenburg MJ, Yoo KH, Bowman LC, Cannon EN, Vanderwert RE (2016). Assessing human mirror activity with EEG mu rhythm: a meta-analysis. Psychol Bull.

[24] Bowman LC, Bakermans-Kranenburg MJ, Yoo KH, Cannon EN, Vanderwert RE, Ferrari PF, et al. The mu-rhythm can mirror: Insights from experimental design, and looking past the controversy. Cortex [Internet]. Elsevier; 2017;96:121–5. Available from: 10.1016/j.cortex.2017.03.025. [cited 2018 May 7]. 10.1016/j.cortex.2017.03.025 28473064

[25] Lepage J-F, Théoret H. EEG evidence for the presence of an action observation-execution matching system in children. Eur J Neurosci [Internet]. 2006;23:2505–10. Available from: http://doi.wiley.com/10.1111/j.1460-9568.2006.04769.x. 10.1111/j.1460-9568.2006.04769.x 16706857

[26] Hunnius S, Bekkering H. What are you doing? How active and observational experience shape infants’ action understanding. Philos Trans R Soc B Biol Sci [Internet]. 2014;369:20130490–20130490. Available from: 10.1098/rstb.2013.0490. 10.1098/rstb.2013.0490 PMC4006192 24778386

[27] Catmur C, Walsh V, Heyes C. Sensorimotor learning configures the human mirror system. Curr Biol [Internet]. 2007;17:1527–31. Available from: 10.1016/j.cub.2007.08.006. [cited 2015 Dec 9]. 10.1016/j.cub.2007.08.006 17716898

[28] Ferrari PF, Tramacere A, Simpson EA, Iriki A (2013). Mirror neurons through the lens of epigenetics. Trends Cogn. Sci.

[29] Wiggett AJ, Hudson M, Tipper SP, Downing PE. Learning associations between action and perception: Effects of incompatible training on body part and spatial priming. Brain Cogn [Internet]. 2011;76:87–96. Available from: 10.1016/j.bandc.2011.02.014. [cited 2017 Jan 20]. 10.1016/j.bandc.2011.02.014 21481998

[30] Heyes C (2010). Where do mirror neurons come from?. Neurosci Biobehav Rev.

[31] Caggiano V, Fogassi L, Rizzolatti G, Pomper JK, Thier P, Giese MA (2011). View-based encoding of actions in mirror neurons of area F5 in macaque premotor cortex. Curr. Biol.

[32] Drew AR, Quandt LC, Marshall PJ (2015). Visual influences on sensorimotor EEG responses during observation of hand actions. Brain Res.

[33] Fu Y, Franz EA. Viewer perspective in the mirroring of actions. Exp Brain Res [Internet]. 2014;232:3665–74. Available from: 10.1007/s00221-014-4042-6. 10.1007/s00221-014-4042-6 25096383

[34] Jackson PL, Meltzoff AN, Decety J (2006). Neural circuits involved in imitation and perspective-taking. NeuroImage.

[35] Frenkel-Toledo S, Bentin S, Perry A, Liebermann DG, Soroker N (2013). Dynamics of the EEG power in the frequency and spatial domains during observation and execution of manual movements. Brain Res.

[36] Kilner JM, Marchant JL, Frith CD (2006). Modulation of the mirror system by social relevance. Soc Cogn Affect Neurosci.

[37] Burgess JD, Arnold SL, Fitzgibbon BM, Fitzgerald PB, Enticott PG. A transcranial magnetic stimulation study of the effect of visual orientation on the putative human mirror neuron system. Front Hum Neurosci [Internet]. 2013;7:Article679. Available from: 10.3389/fnhum.2013.00679. 10.3389/fnhum.2013.00679 PMC3797389 24137125

[38] Blakemore S-J (2008). The social brain in adolescence. Nat Rev Neurosci.

[39] Isoda K, Sueyoshi K, Ikeda Y, Nishimura Y, Hisanaga I, Orlic S, et al. Effect of the hand-omitted tool motion on mu rhythm suppression. Front Hum Neurosci [Internet]. Frontiers; 2016;10:Article 266. Available from: 10.3389/fnhum.2016.00266. [cited 2016 May 26]. 10.3389/fnhum.2016.00266 PMC4889581 27313525

[40] Perani D, Fazio F, Borghese NA, Tettamanti M, Ferrari S, Decety J, et al. Different brain correlates for watching real and virtual hand actions. Neuroimage [Internet]. Affiliation: Institute of Neuroscience and Bioimaging-CNR, Milan, Italy; Affiliation: Scientific Institute H San Raffaele, Milan, Italy; Affiliation: University Vita-Salute, Milan, Italy; Affiliation: University of Milano-Bicocca, Milan, Italy; Affiliatio. 2001;14:749–58. Available from: 10.1006/nimg.2001.0872. 10.1006/nimg.2001.0872 11506547

[41] Muthukumaraswamy SD, Johnson BW, McNair NA (2004). Mu rhythm modulation during observation of an object-directed grasp. Cogn Brain Res.

[42] Oberman LM, Hubbard EM, McCleery JP, Altschuler EL, Ramachandran VS, Pineda JA. EEG evidence for mirror neuron dysfunction in autism spectrum disorders. Cogn Brain Res [Internet]. Affiliation: Center for Brain and Cognition, UC San Diego, San Diego, CA 92093-0109, United States; Affiliation: Department of Psychology, UC San Diego, 9500 Gilman Drive, San Diego, CA 92093-0109, United States; Affiliation: Department of Rehabilitation; 2005;24:190–8. Available from: 10.1016/j.cogbrainres.2005.01.014. [cited 2014 Jul 11]. 10.1016/j.cogbrainres.2005.01.014 15993757

[43] Perry A, Bentin S. Does focusing on hand-grasping intentions modulate electroencephalogram μ and α suppressions? Neuroreport [Internet]. 2010;21:1050–4. Available from: 10.1097/WNR.0b013e32833fcb71. 10.1097/WNR.0b013e32833fcb71 20838261

[44] Stroganova T, Orekhova E, Posikera IN. EEG alpha rhythm in infants. Clin Neurophysiol [Internet]. 1999;110:997–1012. Available from: 10.1016/S1388-2457(98)00009-1. 10.1016/s1388-2457(98)00009-1 10402087

[45] Takemi M, Masakado Y, Liu M, Ushiba J (2013). Event-related desynchronization reflects downregulation of intracortical inhibition in human primary motor cortex. J Neurophysiol.

[46] Woodruff CC, Maaske S (2010). Action execution engages human mirror neuron system more than action observation. Neuroreport.

[47] R Core Team. R: a language and environment for statistical computing. R Foundation for Statistical Computing [Internet]. Vienna, Austria; 2018. Available from: https://www.r-project.org/.

[48] Kuznetsova A, Brockhoff PB, Christensen RHB. lmerTest Package: Tests in Linear Mixed Effects Models. J Stat Softw [Internet]. 2017;82. Available from: 10.18637/jss.v082.i13.

[49] Bates D, Mächler M, Bolker B, Walker S. Fitting Linear Mixed-Effects Models Using lme4. J Stat Softw [Internet]. 2015;67. Available from: 10.18637/jss.v067.i01.

[50] Nakagawa S, Schielzeth H. A general and simple method for obtaining R 2 from generalized linear mixedeffects models. O’Hara RB, editor. Methods Ecol Evol [Internet]. 2013;4:133–42. Available from: 10.1111/j.2041-210x.2012.00261.x. [cited 2018 Apr 21].

[51] Cook R, Press C, Dickinson A, Heyes C. Acquisition of automatic imitation is sensitive to sensorimotor contingency. J Exp Psychol Hum Percept Perform [Internet]. 2010;36:840–52. Available from: 10.1037/a0019256. 10.1037/a0019256 20695703

[52] Cooper RP, Cook R, Dickinson A, Heyes CM (2013). Associative (not Hebbian) learning and the mirror neuron system. Neurosci. Lett.

[53] Chan JSY, Luo Y, Yan JH, Cai L, Peng K. Children’s age modulates the effect of part and whole practice in motor learning. Hum Mov Sci [Internet]. 2015;42:261–72. Available from: 10.1016/j.humov.2015.06.002. [cited 2017 Jan 23]. 10.1016/j.humov.2015.06.002 26112404

[54] Epley N, Morewedge CK, Keysar B (2004). Perspective taking in children and adults: equivalent egocentrism but differential correction. J Exp Soc Psychol.

[55] Surtees ADR, Apperly IA (2012). Egocentrism and automatic perspective taking in children and adults. Child Dev.

[56] Creem SH, Downs TH, Wraga M, Harrington GS, Proffitt DR, Downs JH (2001). An fMRI study of imagined self-rotation. Cogn Affect Behav Neurosci.

[57] Ruby P, Decety J (2001). Effect of subjective perspective taking during simulation of action: a PET investigation of agency. Nat Neurosci.

[58] Wraga M, Shephard JM, Church JA, Inati S, Kosslyn SM (2005). Imagined rotations of self versus objects: an fMRI study. Neuropsychologia.

[59] Eismont E V., Makhin SA, Bakunova A V., Kaida AI, Pavlenko VB. Properties of the EEG μ rhythm and its reactivity during the performance, observation, imitation, and auditory recognition of movements in children aged 4–14 years. Hum Physiol [Internet]. 2017;43:274–9. Available from: 10.1134/S0362119717030057.

[60] Oberman LM, Ramachandran VS, Pineda JA (2008). Modulation of mu suppression in children with autism spectrum disorders in response to familiar or unfamiliar stimuli: the mirror neuron hypothesis. Neuropsychologia.

[61] Raymaekers R, Wiersema JR, Roeyers H. EEG study of the mirror neuron system in children with high functioning autism. Brain Res [Internet]. Elsevier B.V. 2009;1304:113–21. Available from: 10.1016/j.brainres.2009.09.068. [cited 2016 May 6]. 10.1016/j.brainres.2009.09.068 19782668

[62] Miskovic V, Ma X, Chou C-AA, Fan M, Owens M, Sayama H (2015). Developmental changes in spontaneous electrocortical activity and network organization from early to late childhood. Neuroimage.

[63] Muthukumaraswamy SD, Johnson BW. Primary motor cortex activation during action observation revealed by wavelet analysis of the EEG. Clin Neurophysiol [Internet]. 2004;115:1760–6. Available from: 10.1016/j.clinph.2004.03.004. [cited 2015 Oct 5]. 10.1016/j.clinph.2004.03.004 15261854

